# An inter-island comparison of Darwin’s finches reveals the impact of habitat, host phylogeny, and island on the gut microbiome

**DOI:** 10.1371/journal.pone.0226432

**Published:** 2019-12-13

**Authors:** Wesley T. Loo, Rachael Y. Dudaniec, Sonia Kleindorfer, Colleen M. Cavanaugh

**Affiliations:** 1 Department of Organismic and Evolutionary Biology, Harvard University, Cambridge, Massachusetts, United States of America; 2 Department of Biological Sciences, Macquarie University, Sydney, New South Wales, Australia; 3 College of Science and Engineering, Flinders University, Adelaide, South Australia, Australia; 4 Konrad Lorenz Research Center for Behaviour and Cognition and Department of Behavioural Biology, University of Vienna, Vienna, Austria; Museum National d’Histoire Naturelle, FRANCE

## Abstract

Darwin’s finch species in the Galapagos Archipelago are an iconic adaptive radiation that offer a natural experiment to test for the various factors that influence gut microbiome composition. The island of Floreana has the longest history of human settlement within the archipelago and offers an opportunity to compare island and habitat effects on Darwin’s finch microbiomes. In this study, we compare gut microbiomes in Darwin’s finch species on Floreana Island to test for effects of host phylogeny, habitat (lowlands, highlands), and island (Floreana, Santa Cruz). We used 16S rRNA Illumina sequencing of fecal samples to assess the gut microbiome composition of Darwin’s finches, complemented by analyses of stable isotope values and foraging data to provide ecological context to the patterns observed. Overall bacterial composition of the gut microbiome demonstrated co-phylogeny with Floreana hosts, recapitulated the effect of habitat and diet, and showed differences across islands. The finch phylogeny uniquely explained more variation in the microbiome than did foraging data. Finally, there were interaction effects for island × habitat, whereby the same Darwin’s finch species sampled on two islands differed in microbiome for highland samples (highland finches also had different diets across islands) but not lowland samples (lowland finches across islands had comparable diet). Together, these results corroborate the influence of phylogeny, age, diet, and sampling location on microbiome composition and emphasize the necessity for comprehensive sampling given the multiple factors that influence the gut microbiome in Darwin’s finches, and by extension, in animals broadly.

## Introduction

The microbial communities associated with animals, or microbiomes, are now understood to play significant roles in the biology of the host organism. This is perhaps unsurprising given that all metazoans evolved in a microbial world and microorganisms were an essential aspect of the environment in which each species lived [1]. Most microbiome research has focused on its role for human health and disease states [2], [3] as well as studying potential co-evolution between gut microbiomes and host species in mammals [4–6]. In contrast, considerably less attention has been paid to the composition of the microbiome in non-mammalian species.

Birds are a diverse vertebrate clade with unique life history traits compared to mammals, and are widely useful as global indicators of ecosystem health [7]. From the perspective of the microbiome, recent surveys in both old world and new world passerine species have shown the composition of their gut microbiomes to be distinct from mammals in broad taxonomic characterization. Passerine species contain relatively fewer bacteria from the phylum Bacteroidetes and more Proteobacteria, which is opposite to the relative abundance of these bacterial phyla found in mammalian species [8,9]. Correlations between the diversity of bacteria in the microbiome and phylogenetic relationship of the host species have been established; in one case, this signal was found to be stronger than ecological life history traits amongst 51 bird species sampled in the Czech Republic [8]. However, much remains to be learned about the effects of different factors, such as sampling location, diet and host age, on avian microbiome composition.

Changes in the microbiome over the course of development have been documented with great detail in humans, such as tracking individual strains of bacteria between mother and infant [10]. However, relatively few studies have focused on the ontogeny of the gut microbiome in avian species. One study using clone libraries in a member of the gull family, the black legged kittiwake (*Rissa tridactyla*), found a clear separation between nestling and adult cloacal microbiomes with minimal overlap between the operational taxonomic units (OTUs) [11]. Nestlings also appeared to harbor a greater diversity of bacterial taxa based on differences in the rarefaction curves. Another study in chinstrap penguins (*Pygoscelis antarctica*) similarly showed clear clustering by age, but in this case adults had greater inferred richness of bacteria compared to chicks [12]. In two songbird species, the local nest environment was experimentally shown to influence their microbiomes. Using a cross-fostering approach, great tit (*Parus major*) and blue tit (*P*. *caeruleus*) nestlings were swapped between nests; heterospecific nestlings reared in the same nest had closer microbiome communities than conspecific nestlings reared in different nests, pointing to a strong effect of the environment on the assemblage of the microbiome [13].

In considering environmental effects, geographical location is a key variable that may influence the microbiome. In birds, the effect of location was studied in a brood parasitic system using the brown-headed cowbird (*Molothrus ater*) [9]. Cowbird eggs are laid in the nests of heterospecific host species and the cowbird hatchlings are subsequently reared by heterospecific foster adults. By sampling the gut microbiome from cowbird and host species populations in California and Louisiana, Hird *et al*. [14] investigated whether an individual’s gut microbiome would reflect its species identity (e.g., same in cowbirds from California or Louisiana) or that of its foster host species (e.g., different in cowbird reared in host species from California versus Louisiana). The sampling locality had the strongest association with the microbiome composition across all the samples characterized, corroborating a strong environmental effect on the gut microbiome. Another study across the Americas in mammalian species demonstrated a correlation between geographic distance and microbiome distances even after taking the phylogenetic relationships into account [15]. The geographic distance provided additional explanatory power compared to the sole interpretation based on phylogenetic distance. Notably, environmental drivers of microbial community composition are increasingly being found with the emergence of novel genomic and spatial analyses [16].

Darwin’s finches provide an opportunity to compare the effect of locality and phylogeny on gut microbiome in a replicated natural laboratory in the Galápagos Archipelago. With an extensive body of research into the ecology and evolution of these species, each island within the Galapagos archipelago serves as an independent environment to characterize the microbiome in this extremely well-studied adaptive radiation [17]. Previous work has shown the impact of unique diets [18] and the presence of human activity [19] on the gut microbiomes of Darwin’s finch species. However, comprehensive sampling across all species on a single island provide ecological context for observed patterns and such sampling on the island of Santa Cruz documented effects of habitat, foraging behavior, and finch phylogeny on the Darwin’s finch bacterial community [20]. Comprehensive sampling of all extant species from an additional island allows us to test if the patterns hold across islands and test for island-specific effects by comparing species present on both islands.

Floreana Island is located in the south of the archipelago (total area: 173 km2, 1°28’ S, 90°48’ W). Similar to the larger islands in the Galapagos, Floreana Island is characterized by multiple ecological zones ranging from the arid lowlands to the humid highlands. It harbors five extant species of Darwin’s finches: the small ground finch (*Geospiza fuliginosa*), the medium ground finch (*G*. *fortis*), the cactus finch (*G*. *scandens*), the small tree finch (*Camarhynchus parvulus*), and the endemic, critically endangered medium tree finch (*C*. *pauper*) [21]. Within the Galapagos system, Floreana Island has the longest history of human settlement and, likely for this reason, the highest record of local extinction of land birds within the archipelago. The avian species extinction list includes large ground finch (*G*. *magnirostris*) and sharp-beaked ground finch (*G*. *difficilis*) by ~1870 [22], the Floreana mockingbird (*Mimus trifasciatus*) by 1895 [23], the warbler finch (*Certhidea fusca*) by 2004 [24], the large tree finch (*C*. *psittacula*) genetically confirmed absent by 2010 [25], and most recently the vegetarian finch (*Platyspiza crassirostris*), which was not detected in surveys in 2015 [21]. Currently, the impact of the invasive parasitic fly, *Philornis downsi*, which was first discovered in Darwin’s finch nests in 1997 [26], is considered especially detrimental to the persistence of critically endangered species (reviewed in [27]). Floreana Island harbors the only population of the critically endangered medium tree finch [28], which is currently hybridizing with the small tree finch [25,29]. Since 2004, Kleindorfer and colleagues have studied the Floreana Island Darwin’s finch group with insights into nesting behavior [30–33], song [34–36], foraging behavior [37], genetic admixture [29], and hybrid fitness [38]. The hybridization event between the small tree finch and medium tree finch is of particular interest and specific microbiome differences between the genetic clusters is the subject of a separate study [39].

Here we characterized the gut microbiomes of five species of Darwin’s finch found on Floreana Island across both highland and lowland habitats addressing four questions: A) What is the association between Darwin’s finch phylogeny and microbiome community? B) Does age class affect microbiome in small ground finches sampled within the lowlands? C) Do habitat and diet affect finch microbiome? and D) How do microbiome patterns differ between Floreana Island and Santa Cruz Island? Since the species inhabit similar ecological niches on both Floreana Island and Santa Cruz Island, we hypothesized that we would observe similar patterns in the effects of host phylogeny, habitat, and diet. With opportunistic sampling of nestlings, we also examined differences between life stages within the small ground finch. Given that four of our focal Darwin’s finch species occur on both of the islands we examine (Santa Cruz, Floreana), we were able to compare conspecific host microbiomes across islands. Controlling for the effect of species allows us to interrogate whether the island of origin affects the microbiome, in combination with comparisons of foraging patterns and stable isotope analysis as proxies for dietary differences between species and locations. This study leverages the natural, replicated distribution of isolated populations of conspecific Darwin’s finch species to illuminate factors that contribute to gut microbial community structure in birds.

## Materials and methods

### Ethics statement

All samples were collected with permission from the Parque Nacional Galápagos and Ministerio del Ambiente, Ecuador (Research permit No. PC-23-16). All collection protocols were approved by the Institutional Animal Care and Use Committee in the Faculty of Arts and Sciences at Harvard University (Protocol 15-08-249). Samples for the medium tree finch (*Camarhynchus pauper*) were imported under U.S. Fish and Wildlife Service permit number MA05827C-0.

### Study sites and species

Fieldwork was conducted during February 2016 on Floreana Island, Galápagos Archipelago, Ecuador. Sampling sites were located in both highland (1°17’S, 90°27’W) and lowland (1°16’S, 90°29’W) habitats. Five Darwin’s finch species on Floreana Island were sampled and the number of samples per species and per habitat are detailed in Table 1. Samples from two of the ground finch species were primarily collected in the lowlands (L) but also included at least one sample in the highlands (H): the medium ground finch (*Geospiza fortis*) (8L, 2H) and the cactus finch (*Geospiza scandens*) (6L, 1H). The small ground finch included multiple samples from both habitats (15L, 13H), of which three lowland samples were from a set of nestling siblings. All tree finch samples were collected exclusively in the highlands and were previously assigned to genetic population after microsatellite genotyping [39]. After genetic population assignment, there were 4 samples from the medium tree finch (*Camarhynchus pauper*), 14 samples from the small tree finch (*Camarhynchus parvulus*), and 11 samples from the admixed population.

**Table 1:** Darwin’s finch species and sample number from highland and lowland habitats on Floreana Island, Galapagos Archipelago.

Common Name	Abbreviation	Scientific Name	Highland Samples	Lowland Samples	Total per species
Small Ground finch	SGF	*Geospiza fuliginosa*	13	15*	28
Medium Ground finch	MGF	*Geospiza fortis*	2	8	10
Cactus finch	CF	*Geospiza scandens*	1	6	7
Small Tree finch	STF**	*Camarhynchus parvulus*	14		14
Hybrid Tree finch	HTF**	N/A	11		11
Medium Tree finch	MTF**	*Camarhynchus pauper*	4		4
Total			44	30	74

* 3 samples were from small ground finch nestlings

** All tree finches were previously genotyped using microsatellite loci and assigned to genetic clusters [39]

### Sample collection

Finches were caught using mist nets and tagged with an aluminum ring imprinted with a unique identifier prior to all sample collections. Eight morphological measurements were taken for all finches sampled. These included (1) beak-head (beak tip to back of head), (2) beak-naris (beak tip to anterior end of the naris), (3) beak-feather (tip of beak to feather line), (4) beak depth (at the base of the beak), (5) beak width (at the base of the beak), (6) naris diameter (taken from extremes of naris opening), (7) tarsus length, (8) wing length, and (9) body mass. Dial calipers were used to take morphological measurements to the nearest 0.01 mm and Telinga electronic scales were used to measure mass to the nearest 0.01 g. Ground finch individuals were classified into species using the morphological measurements and established protocols [17,25,40], while the tree finches required microsatellite genotyping for accurate classification [20,25,29].

Blood samples were collected for genetic and stable isotope analyses. Samples for genetic analysis were preserved on Whatman FTA Paper (GE Healthcare Life Sciences, Pittsburgh, PA) and stored at room temperature. Samples for stable isotope analysis were dried on small pieces (roughly 0.5 x 0.5 cm^2^) of quartz fiber filter paper (Schleicher and Shuell, Dassel, DE) and stored in microcentrifuge tubes with a silica gel bead as desiccant at room temperature.

After morphological measurements and blood sample collection, fecal samples were collected by placing each finch into a 7" x 7" x 7" cage lined with UV-sterilized parchment paper. Cages were covered with fabric and finches were monitored until defecation for a maximum of 30 min before release. Feces were immediately transferred from parchment paper with bleach cleaned spatulas into pre-weighed microcentrifuge tubes containing 1 ml of DNA/RNA Shield (Zymo Research, Irvine, CA) and mixed by shaking the tubes by hand before storage at -20°C within 4 h of collection to prolong the longevity of the DNA stabilization buffer. The preserved fecal samples were shipped at room temperature and stored at -80°C in the lab until further analysis.

Small ground finch nestling fecal samples were collected directly off the bird bag on which the individuals were placed for weighing. To minimize contamination from the bird bag, only the top layer of the fecal sample was taken, taking care to avoid collecting material in contact with the bird bag.

### Foraging observations

To quantify the diet patterns across species in both habitats, first foraging observations were collected at both highland and lowland sampling sites [41]. At each site, a single walk through of 1 hr was conducted with no overlaps or doubling back to avoid observing the same individuals. During the walkthrough, individual finches were observed until the first food item was ingested. The food item consumed was recorded as one of five categories: insect, seed, flower, leaf, or fruit with the latter four combined as “plant” food items for analyses. Due to the tame nature of Darwin’s finches, the majority of observations were made within 8 m of the focal individual.

### Fecal DNA extraction and 16S rRNA gene sequencing

DNA was extracted from feces in the laboratory using the ZR Fecal Miniprep kit (Zymo Research, Irvine, CA) following manufacturer’s instructions with the following changes. To minimize loss of biological material, BashingBeads were added directly to the collection tubes with the fecal sample in DNA/RNA Shield, which acted as the lysis buffer. Samples were homogenized in a FastPrep FP120 (Qbiogene, Carlsbad, CA) for six rounds of 45 s at speed 6.5 m/s. Between each round, tubes were cooled on ice for 3 min. All liquid transfer steps were performed in a laminar flow hood to minimize environmental contamination.

The V4 region of the 16S rRNA gene was amplified using NEBNext Q5 HotStart HiFi MasterMix 2x (New England Biolabs, Ipswich, MA) and previously designed dual-index barcoded universal primers, which amplify between positions 515F (5’- GTGCCAGCMGCCGCGGTAA-3’) and 806R (5’- GGACTACHVGGGTWTCTAAT -3’) [42]. For each fecal DNA sample, triplicate 25 μl PCR reactions were performed containing 12.5 μl master mix, 9.5 μl molecular grade water, 0.5 μl of 10 M stock for each primer, and 2 μl of DNA template. PCR conditions consisted of initial denaturation at 94°C for 5 min followed by 20 cycles of 98°C for 20 s, 55°C for 15 s, 72°C for 40 s, and a final extension at 72°C for 5 min.

All PCR products were purified using 0.66X Aline PCRClean DX (Aline Biosciences, Woburn, MA) to size select for the ~450 bp PCR product. Purified PCR products were visualized and quantified using High Sensitivity D1000 ScreenTape on an Agilent 2200 TapeStation (Agilent, Santa Clara, CA) and pooled in equimolar concentrations for sequencing on a single MiSeq run (Illumina, USA) using v2 chemistry and 2 x 250-bp paired-end reads at the Harvard Biopolymers Facility (Boston, MA).

#### Contamination controls

Given the low DNA content of bird feces [43], we were concerned about the influence of environmental microbial contamination in analyzing the sequences [44]. To understand the sources of contamination, controls were included at the DNA extraction and amplification steps of the sequencing preparation. To evaluate contaminants from the DNA extraction kits, for each kit we included a mock community extraction with 75 μl of bacterial cells from ZymoBIOMICS Microbial Community Standard (Zymo Research, Irvine, CA) and a no sample extraction control with only DNA/RNA Shield. To assess contaminants from PCR amplification reagents, for each 96-well plate of PCR amplification, a triplicate mock community amplification with 2 μl of a 1:10 dilution of ZymoBIOMICS Microbial Community DNA Standard (Zymo Research, Irvine, CA) and a triplicate no template control reaction. Greater than 99.75% of all reads from ZymoBIOMICS Microbial Community standards and DNA standards mapped to the expected genera. None of the extraction or no template controls produced quantifiable PCR product and were excluded from further analysis.

#### Sequence processing

Sequences were demultiplexed according to the dual-index barcode by the Harvard Biopolymers Facility (Boston, MA) and all the following sequence processing steps were performed in R version 3.4.0 [45]. The fastq files for each sample were converted into Amplicon Sequence Variants (ASVs) using DADA2 with parameters as described in [46]. ASVs were taxonomically classified with the RDP v14 training set [47] and chimeras were removed as implemented in DADA2. After initial processing a total of 3,709,205 reads and 6,015 ASVs were identified across all 74 finch fecal samples.

#### Sequence filtering

The following steps were taken to produce the final dataset for analysis. As it is impossible to prevent all environmental contamination in PCR amplification, the frequency based *decontam* algorithm [48] was applied to the dataset to identify reads from likely contaminants based on the concentration of the PCR products. This removed 22,612 reads (0.61%) and 100 ASVs (1.66%). To reduce the influence of ASVs present in only a few samples, a 5% prevalence filter was applied, which removed 96,837 (2.63%) reads and 2,379 ASVs (40.22%). After taxonomic assignment, any sequences not classified as Bacteria were removed, subtracting 2,334 reads (0.07%) and 24 ASVs (0.68%). Finally, sequences classified as chloroplasts were removed, subtracting 115,724 reads (3.23%) and 36 ASVs (1.03%). The final dataset included 3,471,698 reads and 3,476 ASVs.

#### Rarefying reads

To ensure sample library sizes were not driving the patterns observed in the data, the following categorical variables were checked for significant differences in mean library size (using the Kruskal-Wallis rank sum test) and library size distribution (using Levene’s test as implemented in the R package car [49]): species, habitat, and sex. None of the variables were significantly different in mean library size or library size distribution after Bonferroni correction for multiple comparisons (S1 Table). Therefore, for increased statistical power in detecting differences between microbiome samples, all following analyses were performed with non-rarefied microbiome data [50].

### 16S rRNA sequence analysis of Darwin’s finch microbiomes

#### Alpha diversity analyses

To calculate the relative abundance of bacterial phyla present in the gut microbiome of each Darwin’s finch species, reads were transformed to proportions by sample and then averaged across all microbiome samples per finch species. The alpha diversity metrics (observed ASVs and Chao1) were calculated using phyloseq [51].

#### Beta diversity analyses

To visualize differences between microbiome samples, double principal coordinate analysis (DPCoA) was applied to the log-transformed ASV table as implemented in the R package phyloseq [51]. DPCoA is a dissimilarity metric which incorporates both quantitative and phylogenetic information about the microbiome samples [52]. To assess the differences in community composition of the gut microbiomes between samples, weighted UniFrac distances [53] were calculated between all samples. All abundance data were log transformed prior to distance calculations as an approximate variance stabilization method. To check for the homogeneity of the multivariate dispersions of the distance metrics, the *betadisper* function was used as implemented in the R package vegan [54]. To test the significance of categorical variables, permanova was used as implemented with the function *adonis* in the R package vegan [54].

### Comparative metadata

#### Stable isotope analysis

To assess the differences in diet between the finches sampled, stable isotope analyses were performed using blood samples dried on quartz fiber filter paper. These were packaged in 5 x 9 mm tin capsules for analysis (041077, Costech Analytical Technologies, Inc, Valencia, CA). *δ*^13^C and *δ*^15^N values were measured on a Thermo Scientific Delta V paired with a Costech 4010 elemental analyzer and a high-temperature conversion elemental analyzer at the Center for Stable Isotopes at the University of New Mexico (Albuquerque, NM). Values are expressed in ‰ as *δ* ^13^C = [(R_sample_/R_std_)-1], where R_sample_ = ^13^C/^12^C in a sample, and R_std_ = ^13^C/^12^C in the Vienna Pee Dee Belemnite standard. Similarly, *δ* ^15^N = [(R_sample_/R_std_)-1], where R_sample_ = ^15^N/^14^N in a sample, and R_std_ = ^15^N/^14^N in atmosphere. A known protein standard was run at multiple concentrations as a run-to-run control. *δ*^13^C values were adjusted by the mean difference between the measured values for the protein standard and the known value (-1.18‰). *δ*^15^N values for samples below 1000 mV were error corrected using a linear regression on the protein standard (R^2^ = 0.39).

#### Foraging data

For summarizing foraging data, the food items seed, flower, leaf, and fruit were combined into the category plant. The proportion of plant and insect food items therefore sum to 1. These observations provide knowledge of the broad diet patterns for each finch species in both habitats.

Since few observations were made of cactus finches and medium ground finches in the highlands (zero and one, respectively), samples from these species in the highlands were excluded from any analysis that used foraging data.

### Testing co-diversification of the microbiome and the finch phylogeny

To assess congruence between the phylogenetic diversification of Darwin’s finches and their gut microbiomes, the Procrustean approach to co-phylogeny (PACo) [55] was applied to the data. PACo was designed to detect the similarity of evolutionary patterns in host-parasite associations. Here, the microbiome samples are treated as the ‘parasites’ to compare with the host species genetic distances. Darwin’s finch species’ genetic distances for the PACo analysis were based on whole genome resequencing encompassing more than 44 million variable sites with representative individuals chosen from Santa Cruz when available (Lamichhaney, personal communication [56]. Microbiome distances were calculated using the weighted UniFrac metric [53] to produce a quantitative distance comparison that incorporated the phylogeny of the microbial community. PACo analysis was run as implemented in the R package paco [57], with 10,000 permutations to test the significance of the signal. Using the symmetric calculation, the correlation coefficient r was calculated as r = (1-ss). Because three samples lacked stable isotope data and one sample lacked foraging data, a total of four samples were excluded from PACo analysis with the finch phylogeny.

#### Variation partitioning

To compare the amount of variation explained by host genetic distance, stable isotope values, and diet distance as calculated from first foraging observations, variation partitioning by redundancy analysis [58] was used as implemented with the *varpart* function in the R package vegan [54]. The microbiome distance matrix was used as a response variable with three explanatory tables: the first two principal coordinate axes of the host genetic distance, the *δ*^13^C and *δ*^15^N stable isotope values, and the first two principal component axes of the first foraging observations. Significance of the distance based redundancy analysis was assessed using the *anova*.*cca* function implemented in vegan.

#### Beta diversity through time (BDTT) analysis

To further disentangle the contribution of host phylogeny and diet to the microbiome composition, the beta diversity through time (BDTT) metric [6] was applied to the dataset as described previously [20].

### Inter-island comparison with Santa Cruz data

To investigate whether Darwin’s finch microbiome communities are affected by the island of origin, the samples sequenced in this study were compared with the results from a study of finch species on the island of Santa Cruz [20]. Samples from both studies were collected in the same field season, processed at the same time in lab, and are directly comparable. To specifically test a possible island effect, only the species present on both islands were analyzed for this comparison: the small ground finch, medium ground finch, cactus finch, and small tree finch. To more accurately characterize the microbiomes across islands, only species and habitat combinations with at least three samples were included and a summary table of the combined dataset can be found in S2 Table. Tests of difference in beta diversity by island were performed using PERMANOVA as described above (**Beta diversity analyses**).

To evaluate whether foraging patterns differed between islands, Euclidean distances were calculated between the weighted average foraging pattern for each habitat on both islands. The weighted average foraging pattern was calculated by weighting each food category by the number of samples of each species included in the microbiome comparison.

## Results

Using next-generation sequencing, a total of 3,471,698 sequences were generated across all finch microbiome samples (mean = 31,272; range = 1,792 to 54,585; one outlier with 1,094,988) across a total of 3,476 amplicon sequence variants (ASVs) (mean = 597, range = 43 to 1,396). Sequence numbers were not significantly different across variables of interest and all following analyses are based on the non-rarefied data to increase statistical power (S1 Table; see Methods –Rarefying reads).

### Darwin’s finch microbiome alpha diversity analyses

**Fig 1:**
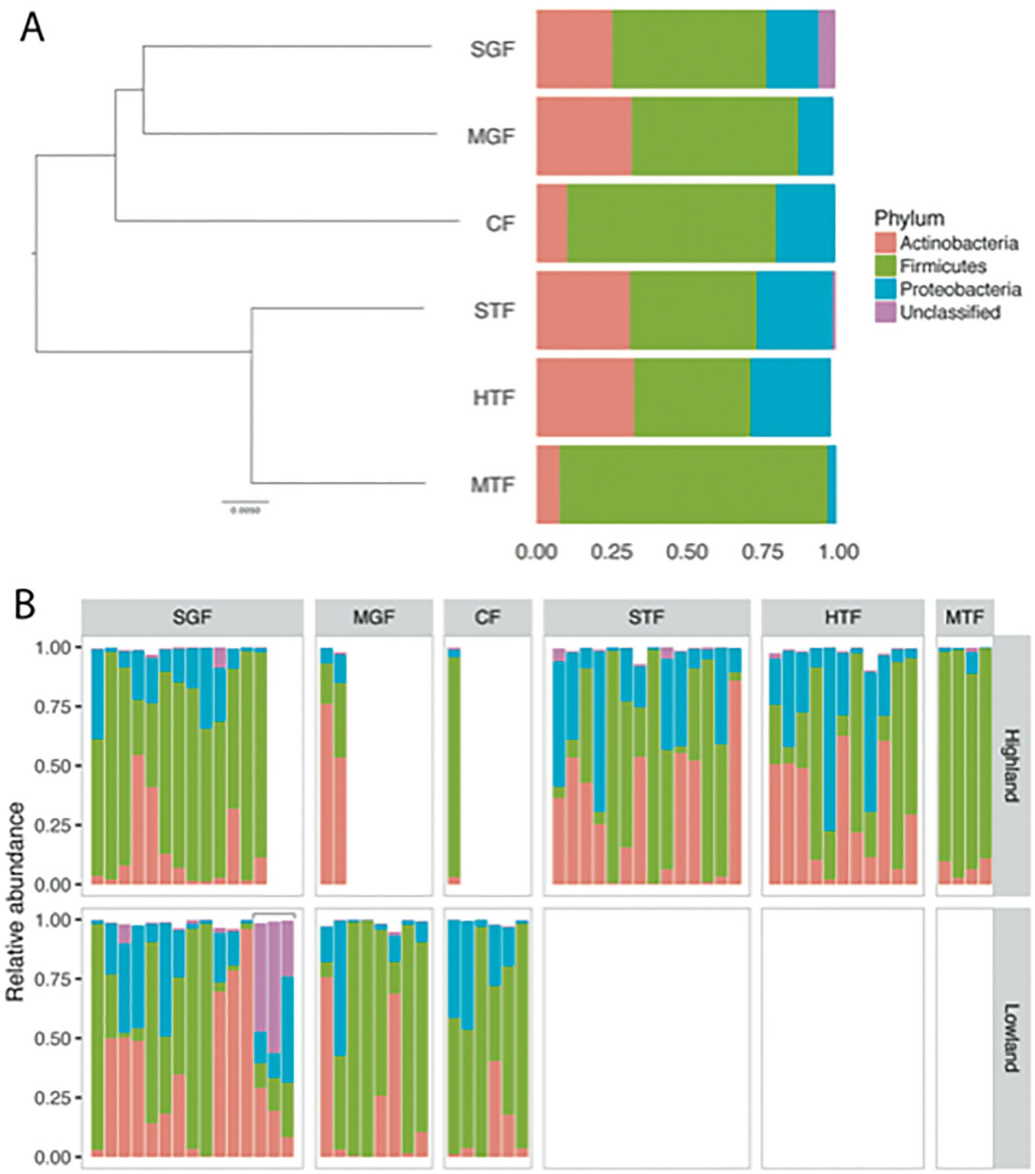
Relative abundance of bacterial phyla in the gut microbiota of Darwin’s finch species on Floreana Island. A) Phylogeny of Darwin’s finch species on Floreana Island based on whole-genome resequencing [56] and the mean relative abundance of bacterial phyla across all gut microbiome samples of each species. Species abbreviations are given in Table 1. (Note: the hybrid cluster was not represented in the whole-genome resequencing dataset and therefore lacks a branch on this phylogeny). B) Relative abundance of the bacterial phyla in individual microbiome samples grouped according to species and habitat. The three nestlings are bracketed within the small ground finch lowland. Any bacterial phylum with mean relative abundance within a given finch species below 1% was omitted from both plots.

A total of nineteen bacterial phyla were detected in the gut microbiome in Darwin’s finches though only four were present at a relative abundance of >1% across all samples. Using DADA2, all sequences were assigned to ASVs and taxonomically classified using the RDP v14 database [47]. Across all samples, the bacterial phyla Firmicutes, Actinobacteria, and Proteobacteria composed the majority of the sequences, representing 51%, 27%, and 19% of ASVs respectively. Bacterial taxa unclassified at the phylum level made up 3% of the sequences while all other bacterial phyla detected were represented by less than 1% of sequences across all samples (S3 Table). Comparing across host species, the medium ground finch and the cactus finch had the highest proportion of Firmicutes (89% and 69% respectively), while the medium ground finch had the highest proportion of Actinobacteria (36%) (Fig 1A; S4 Table). Proteobacteria were most abundant in the small tree finch and the hybrid tree finch genetic cluster at 25 and 27%, respectively.

At lower taxonomic levels, the genus *Lactobacillus* in the phylum Firmicutes was the most abundant bacterial genus across all samples (44%) and within each species (S1 Fig; S5 and S6 Tables). For the medium tree finch, *Lactobacillus* dominated the microbiome, with at least 82% of reads within each individual sample classified to this genus. It was also the most abundant bacterial genus in the other Darwin’s finch species on Floreana Island, ranging from a mean relative abundance of 30%-48% in the hybrid tree finch and cactus finch, respectively. The bacterial genus *Kocuria* in phylum Actinobacteria was the second most abundant bacterial genus in the medium ground finch, small tree finch, and medium tree finch at 10%, 12% and 4%, respectively, and was the third most abundant bacterial genus across all samples at 5% mean relative abundance. The remaining three finch species had different bacterial genera as the second most abundant genus: *Acinetobacter* at 5% in the small ground finch, *Enterococcus* at 13% in the cactus finch, and *Helicobacter* at 7% in the hybrid tree finch (S1 Fig).

The small ground finch provided an opportunity to compare the effect of habitat and age on the composition of the microbiome with multiple samples from the highlands and lowlands and samples from three nestlings. Within samples from the small ground finch, highland samples were characterized by a higher proportion of Firmicutes than lowland samples (70% vs 40%), which were primarily assigned to the genus *Lactobacillus* (65% vs 38% of all reads). In contrast, lowland samples from adult small ground finches had a higher proportion of Actinobacteria (40% vs 14%), which were assigned to many more genera at lower relative abundances (all < 4%; S5 Table). In comparison with adults from the lowland habitat, nestling samples were significantly enriched in bacterial taxa that were unclassified at the phylum level (42% vs 1% in adults; S9 Table; Fig 1B).

The total diversity present in the Darwin’s finch gut microbiome was estimated using two metrics calculated across all samples (mean ± SE): observed ASVs (597.4 ± 36.5) and Chao1 (814.6 ± 42.7). Estimates between species were not significantly different across both alpha diversity measures (S10 and S11 Tables).

### Beta diversity analyses

**Fig 2:**
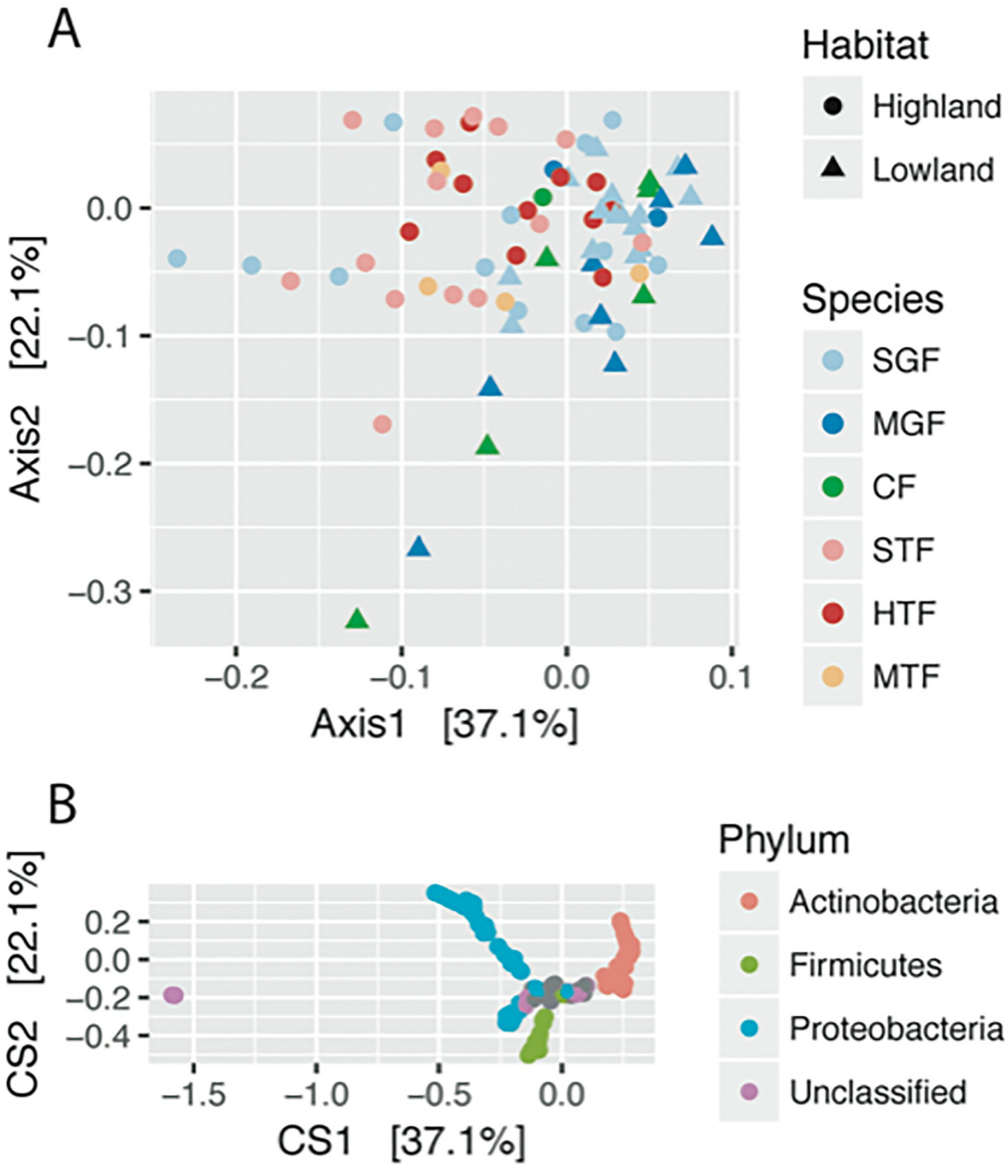
Double principal coordinate analysis (DPCoA) of Darwin’s finch gut microbiome communities. A) Gut microbiome samples from Floreana are plotted on the first two principal coordinate axes, with point color and point shape indicating host species and habitat, respectively. Overall a separation between highland and lowland samples can be seen along the first axis, with highland samples and lowland samples mostly to the left and right, respectively. B) Individual amplicon sequence variants (ASVs) are plotted in the same ordination space as the gut microbiome samples. Bacterial phyla with at least 1% relative abundance across samples are color-coded; all other ASVs are gray. The demarcation between highland and lowland samples is recapitulated by the ASVs, with Proteobacteria and Actinobacteria corresponding to highland (left) and lowland (right) samples, respectively.

To visualize differences in bacterial community composition between microbiome samples, double principal coordinate analysis (DPCoA) was applied to the data. Plotting samples by habitat and species revealed a separation between highland and lowland samples but no clear clustering by species (Fig 2A). The small ground finch was the only species with multiple samples in both habitats and also showed this separation (S2 Fig). By plotting the ASVs in the same ordination space, the bacterial phyla Proteobacteria and Actinobacteria were revealed as enriched in the highland and lowland samples, respectively (Fig 2B). DPCoA did not show visual differences between the adult and nestling small ground finch samples (S3 Fig).

To test whether the differences in beta diversity were statistically significant, the weighted UniFrac distances were tested using PERMANOVA with the categorical variables habitat, species, and sex. Habitat and species were tested in a two-way PERMANOVA to account for the presence of the small ground finch in both habitats. Age was tested within small ground finch samples collected in the lowland. Only habitat showed a significant difference between microbiome communities (R^2^ = 0.10, p = 0.001; S12 Table).

### Stable isotope values and foraging data

**Fig 3:**
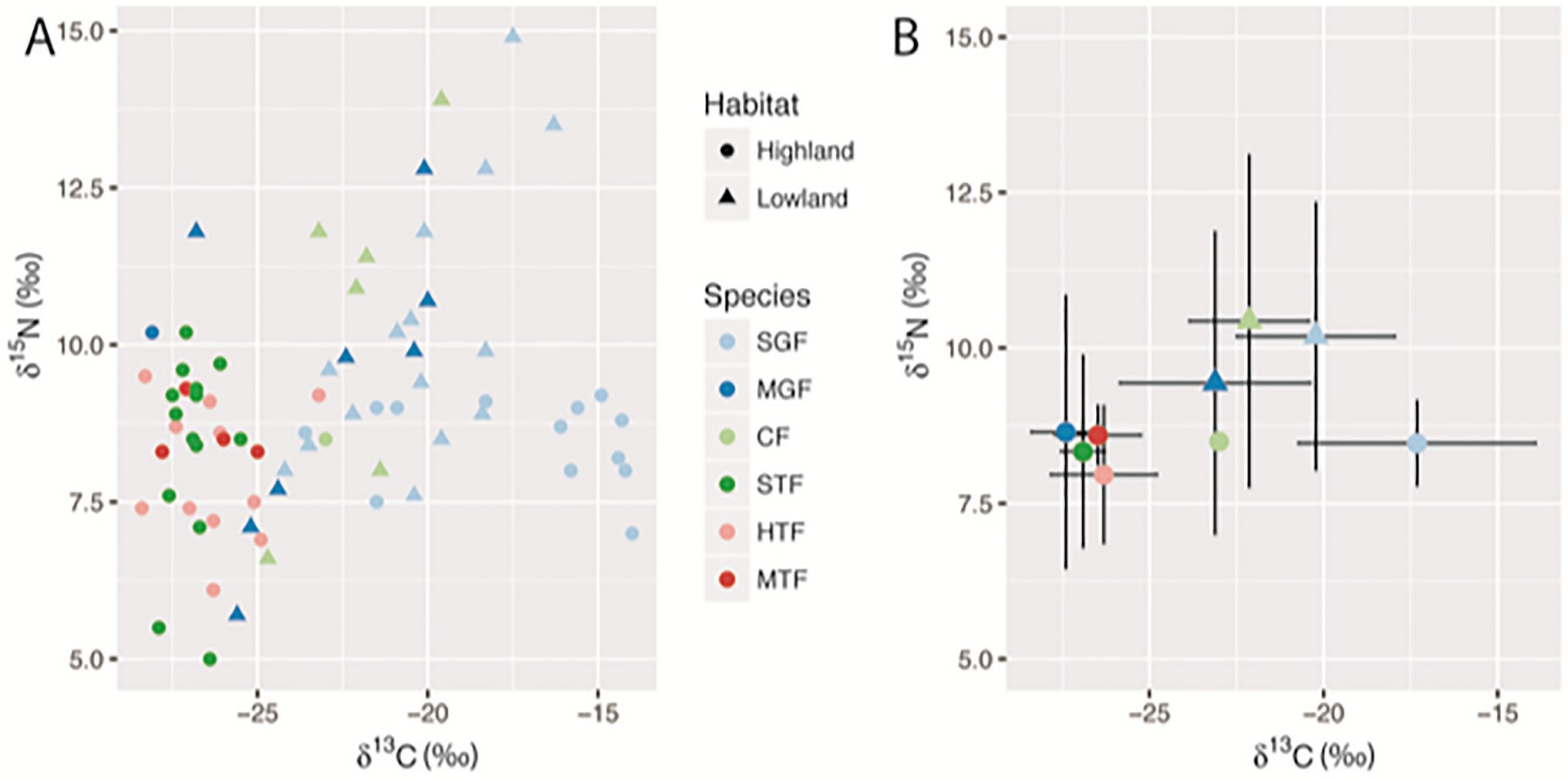
*δ*^13^C and *δ*^15^N stable isotope measurements for Darwin’s finch species. Point color and shape indicate host species and habitat, respectively. A) Individual *δ*^13^C and *δ*^15^N values for each finch with gut microbiome samples. B) Mean *δ*^13^C and *δ*^15^N values for each species and habitat with standard deviation. The ground finch species (SGF, MGF, and CF) all had at least one sample in both habitats and have distinct *δ*^13^C and *δ*^15^N values dependent on the habitat of origin.

To estimate the dietary differences between individuals sampled for the microbiome, *δ*^13^C and *δ*^15^N stable isotope ratios were analyzed. In general, the samples separated more by habitat than by species with highland samples generally lighter in both ^13^C and ^15^N (Fig 3; S13 Table). The tree finch samples in the highland ranged from *δ*^13^C and *δ*^15^N values of -22.8‰ to -28‰ and 5.0‰ to 10.0‰, respectively. In contrast, the lowland samples across the ground finch species (SGF, MGF, CF) had a wider range in both elements, with *δ*^13^C from -26.8‰ to -16.3‰ and *δ*^15^N from 5.7‰ to 14.9‰. Small ground finch samples in the highland had the heaviest *δ*^13^C values around 15‰.

To assess the microbiome samples and stable isotope values in the context of food items, foraging observations were made in both habitats (Table 2). By classifying the observations for each species, it is possible to get a sense of the broad dietary patterns. Since species classification was made visually, observations for the small tree finch and the hybrid cluster were combined. The tree finch species (STF/HTF and MTF) primarily consumed insects, though the medium tree finch also consumed 11% seed. The cactus finch exclusively foraged on plant material though this was spread between flower, leaf, and seed items. The medium and small ground finches consumed both plant material and insects with an increasing proportion of the latter in the highlands.

**Table 2:** First foraging observations across all Darwin’s finch species on Floreana Island.

Species	Habitat	Total (n)	Counts (% of counts)					Summary %	
			Flower	Fruit	Leaf	Seed	Insect	Plant*	Insect
SGF	L	60	22 (36)		4 (7)	24 (40)	10 (17)	83	17
	H	49	1 (2)			26 (53)	22 (45)	55	45
MGF	L	30	1 (3)		11 (37)	12 (40)	6 (20)	80	20
CF	L	20	12 (60)		7 (35)	1 (5)		100	
STF/HTF	H	23					23 (100)		100
MTF	H	19				2 (11)	17 (89)	11	89

* The category ‘Plant’ is the sum of all plant derived food items (flower, fruit, leaf, and seed).

### Testing co-diversification with Floreana species

**Fig 4:**
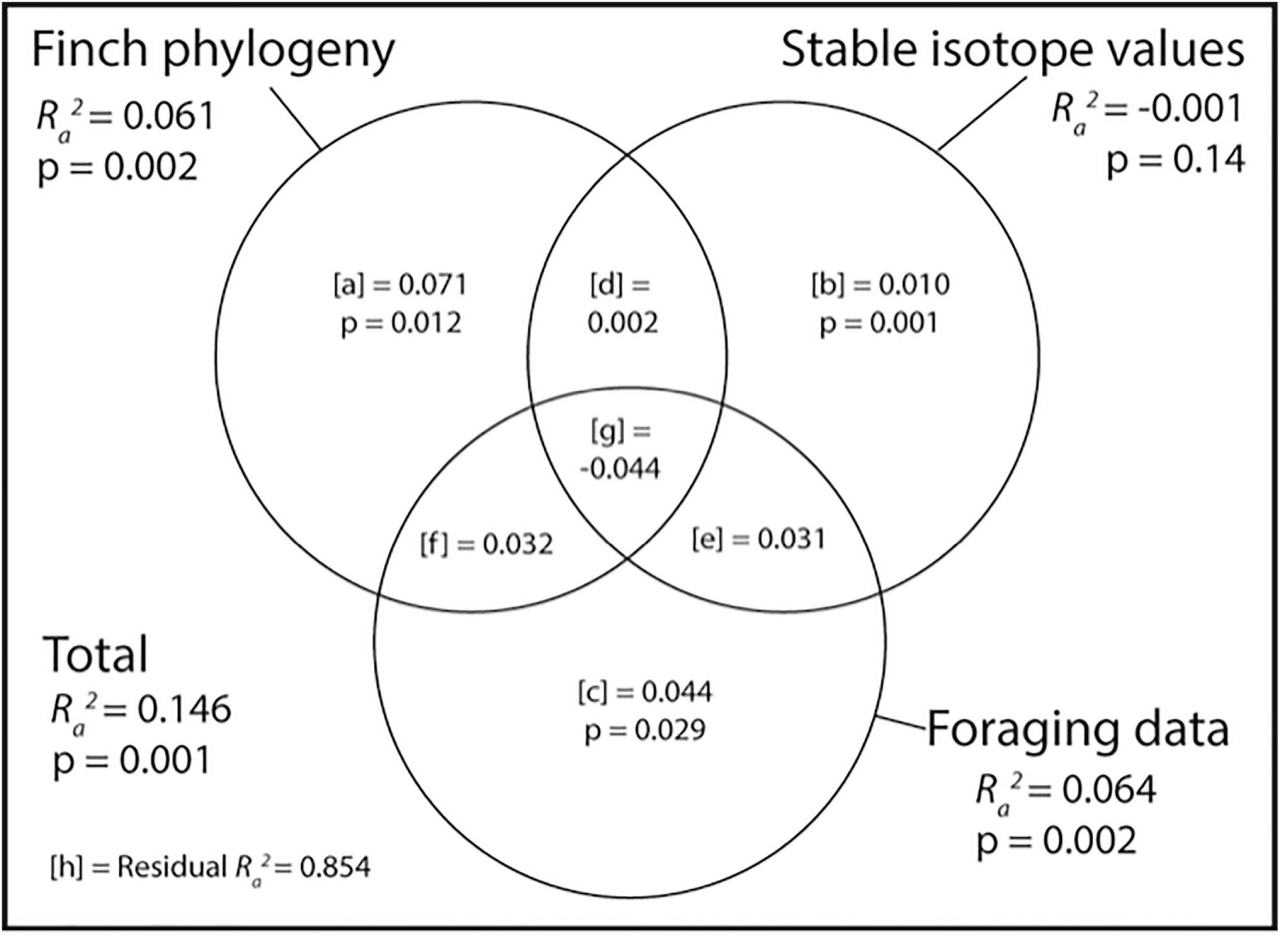
Variation partitioning results for comparing Darwin’s finch phylogeny, stable isotope values, and foraging data to the weighted UniFrac distances of gut microbiome samples from Floreana. Results of variation partitioning using weighted UniFrac distances between gut microbiome samples against Darwin’s finch phylogeny (first two principal coordinate axes), stable isotope values (*δ*^13^C and *δ*^15^N values), and foraging data (first two principal component axes) visualized with a Venn diagram. Adjusted R^2^ values for each component are plotted inside the circles. All testable components include the p-value calculated using distance based redundancy analysis. Adjusted R^2^ values in [a], [b], and [c] are the amount of variation explained uniquely by the corresponding explanatory table. Parts [d], [e], and [f] are amounts of variation that can be explained by either table in the overlap and part [g] is shared by all three tables. Foraging data is the only table with a positive adjusted R^2^ value after controlling for overlapping variance.

PACo analysis showed significant correlation between the host phylogeny and the microbiome (R^2^ = 0.11, p = 0.002). PACo analysis was also applied to the stable isotope and foraging data. Foraging data had a similar procrustean correlation coefficient (R^2^ = 0.12, p < 0.001) while the correlation with stable isotope values was not significant (R^2^ = 0.03, p = 0.25). Variation partitioning was used to compare the amount of variance in the microbiome explained by the finch phylogeny, stable isotope values, and foraging data. Total explained variance with all three explanatory tables had an adjusted R^2^ = 0.15 (Fig 4). The finch phylogeny and foraging data had comparable correlations with the microbiome samples (adjusted R^2^ = 0.06, p = 0.002 for both) while stable isotope values were not significant (adjusted R^2^ = -0.001, p = 0.14). After controlling for variation explained by the overlap between explanatory tables, the finch phylogeny uniquely explained more variation in the microbiome than foraging data (adjusted R^2^ = 0.071 v 0.044, p = 0.012 v 0.029, respectively).

To further parse the correlation between gut microbiome and explanatory variables, the beta diversity through time (BDTT) was applied to the Floreana samples. There was no significant correlation between gut microbiome distance and explanatory variables at any time points (S5 Fig).

### Inter-island comparison with Santa Cruz

**Fig 5:**
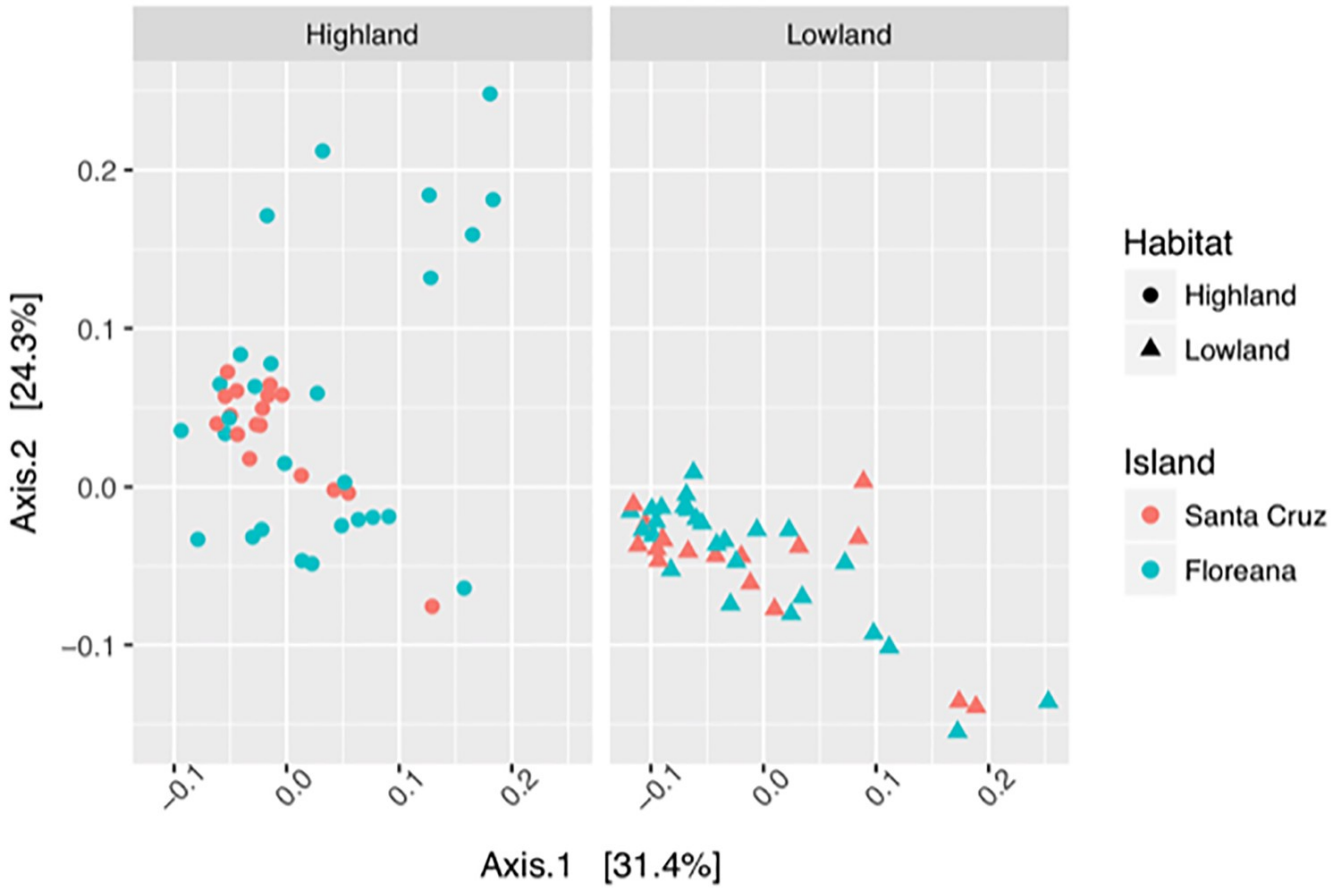
Principal coordinate analysis visualization of weighted UniFrac distances between microbiome samples from Santa Cruz and Floreana Islands. The four species found on both islands (SGF, MGF, CF, and STF) are plotted with shape and color representing the habitat and island of origin, respectively. This is a single PCoA plot faceted by habitat.

To evaluate whether island of origin affects the gut microbiome composition of Darwin’s finches, data from this study were analyzed in conjunction with samples from Santa Cruz Island for the species that occur on both islands: small ground finch (Floreana: 25, Santa Cruz: 13), medium ground finch (F: 8, S: 6), cactus finch (F: 6, S: 6), and small tree finch (F: 14, S: 9), totaling 87 fecal microbiome samples [59]; S2 Table). Though visualization with DPCoA did not reveal strong clustering by island (Fig 5), when tested with a two-way PERMANOVA, both habitat and the interaction term island × habitat were significantly different in weighted UniFrac distances (p = 0.001 and 0.048, respectively) while species nested within habitat or island were not (p = 0.202 and 0.064, respectively; S14 Table). To further determine which combinations of island × habitat were contributing to the difference, pairwise tests were performed in each category. After correcting for multiple hypothesis testing, three of the four pairwise tests were significant–highland on Floreana versus highland on Santa Cruz (R^2^ = 0.07, p = 0.008), lowland versus highland on Santa Cruz (R^2^ = 0.19, p = 0.001), and lowland versus highland on Floreana (R^2^ = 0.13, p = 0.001) (S15 Table). The lowland comparison between islands was not significantly different in weighted UniFrac distances (R^2^ = 0.02, p = 0.431).

To check whether foraging patterns may explain the difference in microbiome samples between the highland habitats, pairwise Euclidean distances were calculated between the weighted average foraging pattern in each island and habitat combination. Euclidean distances between habitats within each island (0.71 and 0.63 for Floreana and Santa Cruz, respectively), were higher than the differences between islands within habitats (0.16 and 0.05 for highland and lowland, respectively; S16 Table). This pattern held even after collapsing the four plant based food items (seed, leaf, flower, fruit) into a single ‘plant’ category. Comparing the proportion of observed food items consumed in the highlands between islands, the small ground finch on Floreana had a higher proportion of insect consumption compared to Santa Cruz (45% v 18%, respectively). The small tree finch was only observed to eat insects on Floreana while on Santa Cruz it consumed a small proportion of seeds as well (10%).

## Discussion

We build on previous microbiome research into nine extant Darwin’s finch species on Santa Cruz Island and, using analysis of the 16S rRNA sequences from an additional 74 finches on Floreana Island, compare the microbiome in five extant species on Floreana Island to study the effects of: (1) phylogeny, (2) age class (nestlings, adults), (3) habitat (lowlands, highlands), and (4) island (Floreana, Santa Cruz). Overall, the composition and diversity observed in the samples from Floreana Island were congruent with findings from other avian studies. Interrogating host phylogeny, Procrustes Analysis of Co-phylogeny revealed a significant correlation between the host genetic distance and microbiome community. However, compared with Darwin’s finch microbiome research from Santa Cruz Island, Beta Diversity Through Time analysis did not recapitulate the correlation between phylogeny and microbiome on Floreana Island. Nestlings had more unclassified bacterial phyla than adults, but did not appear to harbor phylogenetically distinct bacterial taxa. Within Floreana samples, habitat and diet showed significant effects on the microbiome. Finally, a comparison between the microbiome of species that occur on both Santa Cruz and Floreana Islands showed an interaction effect between island × habitat: the microbiome differed for species sampled in the highlands of each island, but did not differ for species sampled in the lowlands. This pattern was congruent with observations on foraging behavior and stable isotope analysis. Highland foraging behavior differed across the two islands, whereas lowland foraging behavior was comparable. Similarly, stable isotope values had larger differences between the highlands of each island compared to the lowlands.

### Composition and diversity of Darwin’s finch microbiomes on Floreana

The gut microbiomes of Darwin’s finch species from Floreana Island are broadly similar to previously characterized avian microbiomes. The bacterial phyla Firmicutes, Actinobacteria, and Proteobacteria comprise the majority of sequences across five Darwin’s finch species, with no other phyla rising above 1% in mean relative abundance. These three phyla are well represented in surveys of broader bird species and were also dominant in the nine finch species from Santa Cruz; however, on Santa Cruz Island, the phyla Bacteroidetes, Chloroflexi, and Tenericutes also rose above this threshold [8,9,59]. At lower taxonomic levels, the genus *Lactobacillus* dominates the microbiome in the five species sampled, ranging from 30% to 89% in the hybrid tree finch and medium tree finch, respectively. The prevalence of *Lactobacillus* was previously noted in the gut microbiomes of four Darwin’s finch species that occur on both Floreana Island and Santa Cruz Island, namely small ground finch, medium ground finch, cactus finch, and small tree finch[59].

### Nestlings harbor unclassified bacteria at higher abundances

The gut microbiome samples from small ground finch nestlings offered an opportunity to investigate age related differences in Darwin’s finches. The comparison of the nestlings to small ground finch adults within the lowlands revealed a much higher proportion of unclassified bacterial taxa at the phylum level, controlling for host species and habitat effects (Fig 1). However, DPCoA revealed no difference in nestling and adult small ground finches (S3 Fig). DPCoA calculates distances between microbial communities that incorporate the phylogenetic structure of the bacterial taxa. Therefore, the lack of significant differences in beta diversity between adults and nestlings implies that while the enriched bacterial taxa are unclassified, they are not phylogenetically distinct. Given the small sample size (n = 3) and the fact that the samples were collected from the same nest, this study only serves as anecdotal evidence for similarity between nestling and adult microbiomes. The lack of clear clustering by age is different than other bird species in which nestling to adult comparisons have been made, such as the black legged kittiwake and the chinstrap penguin, both of which showed a distinct microbial community in the nestlings [11,12]. Further investigation is needed to test whether nestlings have a distinct gut microbiome compared to adults.

### Habitat effects in the context of foraging and stable isotope analysis

In beta diversity tests of habitat, species, and age, only habitat showed a significant difference in the microbiome community. On Floreana Island, only the small ground finch was present at high enough densities for multiple samples in both the humid highlands and arid lowlands. The ground finches (medium ground finch and cactus finch) were primarily sampled in the lowlands and the tree finches (small, medium, and hybrid tree finch) were sampled in the highlands. Though the correlation between the close phylogenetic relationships of species sampled in each habitat leaves open the possibility that host phylogeny explains the difference, visualization of the samples with DPCoA shows a shift in the microbiome community even within the small ground finch (S2 Fig). Additionally, similar analysis run on the communities on Santa Cruz Island showed the same habitat effect after including two phylogenetically more distant species, the vegetarian finch and the warbler finch [59]. Therefore, it is likely that the habitat effect is not due solely to the phylogenetic relationship between Darwin’s finch species.

Foraging data provide context to the observed differences in gut microbiomes between habitats. The observations analyzed in this study across five food categories indicate differences in foraging behavior across species and habitats. Given the short time frame for foraging observations in this study, it is possible that Darwin’s finches were foraging opportunistically in relation to easily accessible food items during the early wet season. In the lowlands, both the cactus finch and medium ground finch were only observed foraging on plant material. The small ground finch was observed in both the lowlands and highlands, and had markedly more insect consumption in the highlands. This finding of greater invertebrate diet in the highlands was also found in a study into small ground finch foraging behavior on Santa Cruz Island [41]. Both tree finch species observed in the highlands largely overlapped in foraging behavior and diet; they mostly consumed insects. Notably, medium tree finches consumed more seeds than small tree finch in this study. Previous research has documented different foraging behaviors between the tree finches on Floreana Island, with more chipping, prying and biting by foraging medium tree finch compared with more gleaning and probing from leaves by foraging small tree finch [37]. In conclusion, the dietary observations from this study align with those from previous studies and point to species and habitat differences in foraging behavior. The dietary observations were done on a random sample of birds in the field that were not subsequently sampled for microbiome characterization, and thus while they cannot align directly with the microbiome characterization, they are an important consideration in interpreting the analyses of the microbiome.

*δ*^13^C and *δ*^15^N stable isotope ratios are representative of the diet of the individual sampled and have been used to characterize differences in diets and trophic partitioning [60,61]. The measured stable isotope values from Floreana Island were partitioned by habitat of origin, with lowland samples generally heavier in both *δ*^13^C and *δ*^15^N. The exception to this pattern was the small ground finch in the highlands, which had eight samples with *δ*^13^C values around -15‰. These are likely driven by the consumption of seeds from a grass species within the genus *Paspalum* (SK, personal observations), which are known to use the C4-carbon fixation pathway and therefore have a *δ*^13^C ratio between -12 and -15‰ [62,63]. Speaking to the broader dietary habits of this species, the small ground finch also had largest range in *δ*^13^C measurements, with other highland individuals at -24‰. The general utility of *δ*^15^N values is in determining trophic levels between organisms, with an average enrichment of ~3‰ per trophic level [64]. Within the primarily insectivorous tree finches, the *δ*^15^N range from 5–10‰ suggests the possibility of the consumption of insects at different trophic levels. The wider range of lowland samples from 6–15‰ is less obvious given the observed primary consumption of plant material. The interpretation of these values is difficult without comprehensive sampling of potential food sources.

Both foraging data and stable isotopes were used as alternative explanatory variables in testing for correlation between the gut microbiome and the finch phylogeny. Two methods of correlating the microbiome samples and the metadata, Procrustes Analysis of Co-phylogeny and variation partitioning, found significant correlation for both finch phylogeny and foraging data, but not for stable isotope values. However, the Beta Diversity Through Time analysis did not show correlation for any of the three metadata variables with the gut microbiome, in contrast with previous characterization of Darwin’s finch microbiomes on Santa Cruz that found significant correlation using all three methods [59]. Two possible reasons for the discrepancy between these methods and previous results are, first, the sample size for the tests are different–BDTT requires a single microbiome per species, which collapses the samples into five data points, whereas both PACo and variation partitioning are run on individual samples. Second, Santa Cruz tested nine species compared to five on Floreana, with the added difference in the phylogenetic structure of the represented finch species. Santa Cruz included two phylogenetically distinct species, the vegetarian finch and the warbler finch, which may provide a larger portion of the phylogenetic signal. The results of these tests on Floreana are therefore consistent with the results from Santa Cruz, taking into account the change in sample size for the BDTT calculation.

### Inter-island comparison shows a convergence in lowland but not highland microbiome samples

The characterization of Darwin’s finch gut microbiomes from Floreana not only provides an independent case study of previous patterns, but also provides a unique opportunity to investigate the effects of habitat and diet alone by controlling for the host species. Four species of Darwin’s finches were sampled on both islands: the small ground finch, medium ground finch, cactus finch, and small tree finch. Additionally, the small ground finch was sampled in all four habitat and island combinations. The samples collected on each island can be treated as independent because the probability of cross-island migration between Santa Cruz and Floreana islands is exceedingly low: in the four decades of work on Daphne Island (with over 90% of individuals tagged), only one immigrant finch from Santa Cruz was detected [65]. While Daphne and Santa Cruz are ~8 km apart, Floreana and Santa Cruz are an order of magnitude farther (~75 km), making it unlikely that any individuals sampled came from the other island.

Testing for an island effect using pairwise comparison of habitat and island combinations showed significant differences between habitats on each island and between the highlands of each island, but not the lowlands. The difference between the highlands had a small effect size, but was still significant. Differences in overall foraging patterns between islands and habitats were calculated using a weighted average across the individuals represented with microbiome samples. These differences were highest between highland and lowland habitats on each island and between the highlands of Santa Cruz and Floreana but much smaller between the lowlands, which is consistent with the differences seen in the microbiome, suggesting that foraging is a primary factor in microbiome composition.

In addition to different foraging behavior of the finch species per habitat and island, there are also expected differences in the distribution and abundance of flora and fauna across islands [66]. To our knowledge, there is no species list for the highland and lowland plants on Floreana Island in particular. The impact of human history has also been different on the inhabited islands, and likely resulted in different agricultural practices, introduced crops, and invasive weeds that have yet to be formally described. For example, though both Santa Cruz and Floreana highlands are dominated by *Scalesia pedunculata*, it has been noted that the *Scalesia* forests on Floreana are unlikely representative of undisturbed vegetation, in part due to the island having the longest history of human habitation within the archipelago [67].

The divergence in finch gut microbiome communities we observe across islands highlights the role of dispersal limitation in determining microbial community structure within wild host species (reviewed in [16]). Previous work has shown that geographic distance increased the distance between gut microbiomes in mammalian species, tested in 17 species across the Americas [15]. However, the distances tested in that study were orders of magnitude larger than the geographic separation between Santa Cruz and Floreana island, with pairs of species up to 11,000 km apart compared to the roughly 75 km between islands. The similarity of microbiomes in lowland finches implies that the bacteria observed are not limited to single islands in their distribution. Therefore, the differences seen between highland samples across islands must be attributed to factors other than geographic distance alone. This pattern is also consistent with other work that found sampling locality to be the most detectable signal in a brood parasite and its host species [14]. There, the cowbird species did not show a species-specific signal but clustered by geographic location. Given the detectable difference between highland, but not lowland, habitats on both islands in Darwin’s finches, it is possible that avian microbiomes reflect the ecological environment in which they reside. Taken together, the inter-island comparison shows the importance of comprehensive sampling of multiple individuals in all habitat/islands of comparison due to the significant effects of both these variables on microbiome composition. Notably, our results suggest that analyses of host species effects on the microbiome of avian species should take geographic location into account, given the contrasting habitats which many conspecific birds occupy.

### Conclusion

Our study further resolves the factors that affect microbiome composition in Darwin’s finches within and across islands of the Galapagos, revealing potential drivers of host-microbial co-evolutionary patterns in this iconic adaptive radiation. Findings from the five species characterized on Floreana island recapitulate many of the broad patterns observed from Santa Cruz and provide an independent sampling event to interrogate the interplay between island and habitat. The difference in gut bacterial community observed between the highlands of Santa Cruz and Floreana demonstrate a clear environmental effect independent of host species and show that foraging habits play a critical role in determining the composition of the gut microbiome. Given the importance of dietary niches in the diversification of Darwin’s finches, our results emphasize the importance of the microbiome in the ecology and evolution of species within this adaptive radiation.

## Supporting information

S1 Fig Mean relative abundance of bacterial genera in Darwin’s finch microbiome samples from Floreana. Only bacterial genera with mean relative abundance greater than 5% for a given finch species is shown.
(PDF)

S2 Fig Double principal coordinate analysis of Darwin’s finch microbiome samples faceted by species. Sample shape and color correspond to habitat and species, respectively.
(PDF)

S3 Fig DPCoA plot of small ground finch microbiome samples from the lowlands with adults vs nestlings. The three nestling samples are tightly clustered but not differentiable from the adults (PERMANOVA with weighted UniFrac p = 0.17).
(PDF)

S4 Fig *δ*^13^C and *δ*^15^N stable isotope measurements for Darwin’s finch species across Santa Cruz and Floreana islands. Point color and point shape indicate host species and habitat, respectively. The four species that are present on both islands are plotted. A) Individual *δ*^13^C and *δ*^15^N values for each finch with gut microbiome samples. B) Mean *δ*^13^C and *δ*^15^N values for each species and habitat with standard deviation. Three points were the only sample from that habitat and species combination and therefore lack standard deviation error bars: the medium ground finch in the highlands and the small tree finch in the lowlands on Santa Cruz in addition to the cactus finch in the highlands on Floreana.
(PDF)

S5 Fig Beta diversity through time applied on the gut microbiome data. Lines show pairwise Sorensen dissimilarities of Darwin’s finch gut microbiome samples determined by time slices every 10 Mya correlated to pairwise dietary distances calculated with first foraging observations (red), pairwise dietary distances using *δ*^13^C and *δ*^15^N stable isotope measurements (green), and pairwise host phylogenetic distances (blue). Correlation did not go above 0.15 for any of the three layers of metadata.
(PDF)

S1 Table Statistical tests on amplicon library size mean and distribution across categorical variables of interest. (PDF)

S2 Table Summary of samples used for inter-island comparison. (PDF)

S3 Table Relative abundance of bacterial phyla across all samples. (PDF)

S4 Table Relative abundance of bacterial phyla by Darwin finch species on Floreana Island. (PDF)

S5 Table The relative abundance (%) of the most abundant bacterial genera across all Darwin finch gut microbiome samples from Floreana. (PDF)

S6 Table Relative abundance (%) of the most abundant bacterial genus in each species of Darwin’s finches on Floreana. (PDF)

S7 Table Relative abundance (%) of the second most abundant bacterial genus in each species of Darwin’s finches on Floreana. (PDF)

S8 Table Relative abundance of bacterial taxa in small ground finch samples from highland and lowland habitats on Floreana. (PDF)

S9 Table Relative abundance of bacterial taxa in the small ground finch adults and nestlings from Floreana Island. (PDF)

S10 Table Alpha diversity estimates by Darwin’s finch species on Floreana. (PDF)

S11 Table Anova on alpha diversity metrics across species (N = 6, sample size ranging from 4 to 28). (PDF)

S12 Table Permanova tests of weighted UniFrac distances with categorical variables of interest. +Age was tested using only small ground finch (*G*. *fuliginosa*) samples collected in the lowland (Adults = 12, Nestling = 3).
(PDF)

S13 Table Stable isotope (*δ* ^13^C and *δ* ^15^N) ratios by Darwin’s finch species and habitat on Floreana. * Single sample for this species/habitat so standard deviation was not calculated.
(PDF)

S14 Table PERMANOVA results for combined dataset across Santa Cruz and Floreana Island with weighted UniFrac distances. (PDF)

S15 Table Post hoc pairwise comparisons of island and habitat combinations with weighted UniFrac distances. (PDF)

S16 Table Pairwise Euclidean distances between weighted average foraging patterns in each island/habitat combination for all five food categories (lower triangle) or broad plant v insect food categories (upper triangle). (PDF)
