# Supplementary material for: An inter-island comparison of Darwin’s finches reveals the impact of habitat, host phylogeny, and island on the gut microbiome
Source: PLoS One. 2019 Dec 13;14(12):e0226432. doi: 10.1371/journal.pone.0226432 (PMC6910665; doi:10.1371/journal.pone.0226432)
Supplement: S1 Fig — Only bacterial genera with mean relative abundance greater than 5% for a given finch species is shown. (PDF) [file pone.0226432.s001.pdf]

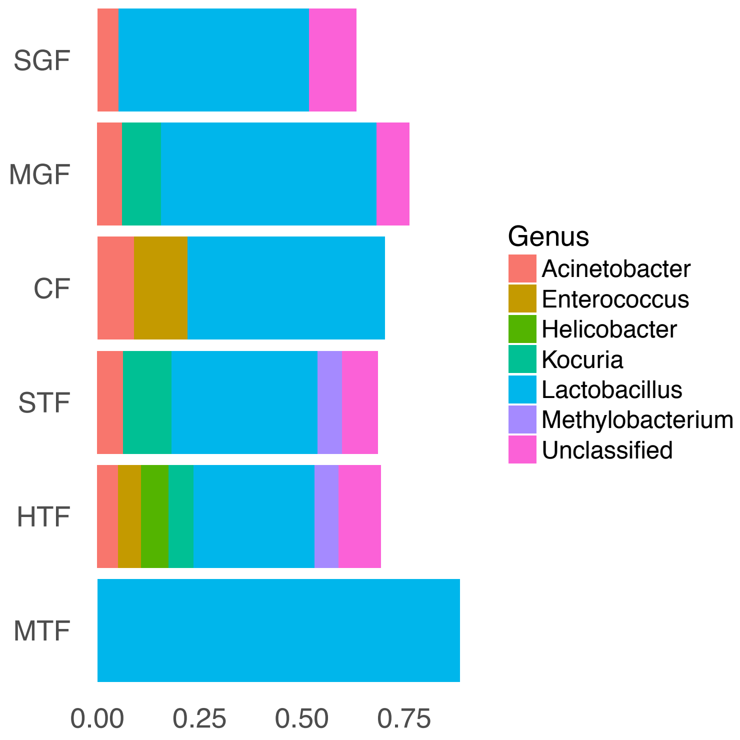

S1 Fig. Mean relative abundance of bacterial genera in Darwin's finch microbiome samples from Floreana.

Only bacterial genera with mean relative abundance greater than 5% for a given finch species is shown.
