# Supplementary material for: An inter-island comparison of Darwin’s finches reveals the impact of habitat, host phylogeny, and island on the gut microbiome
Source: PLoS One. 2019 Dec 13;14(12):e0226432. doi: 10.1371/journal.pone.0226432 (PMC6910665; doi:10.1371/journal.pone.0226432)
Supplement: S2 Fig — Sample shape and color correspond to habitat and species, respectively. (PDF) [file pone.0226432.s002.pdf]

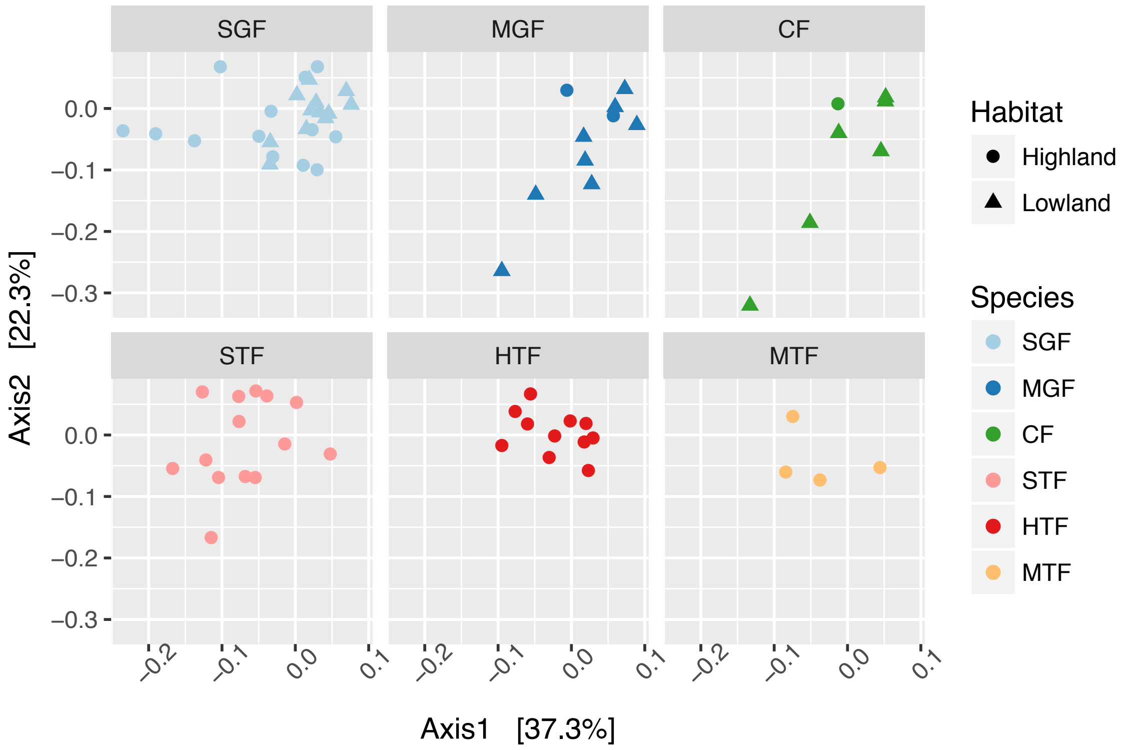

S2 Fig. Double principal coordinate analysis of Darwin's finch microbiome samples faceted by species.

Sample shape and color correspond to habitat and species, respectively.
