# Supplementary material for: An inter-island comparison of Darwin’s finches reveals the impact of habitat, host phylogeny, and island on the gut microbiome
Source: PLoS One. 2019 Dec 13;14(12):e0226432. doi: 10.1371/journal.pone.0226432 (PMC6910665; doi:10.1371/journal.pone.0226432)
Supplement: S3 Fig — The three nestling samples are tightly clustered but not differentiable from the adults (PERMANOVA with weighted UniFrac p = 0.17). (PDF) [file pone.0226432.s003.pdf]

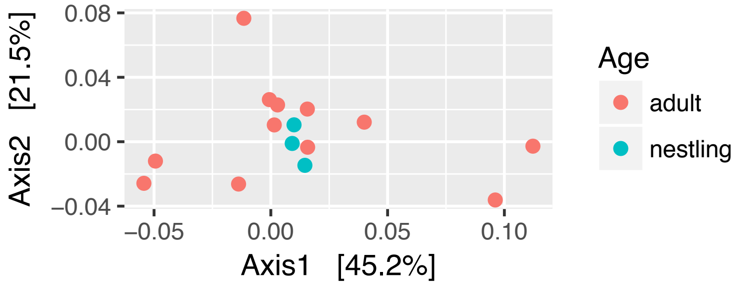

S3 Fig. DPCoA plot of small ground finch microbiome samples from the lowlands with adults vs nestlings.

The three nestling samples are tightly clustered but not differentiable from the adults (PERMANOVA with weighted UniFrac p=0.17).
