# Supplementary material for: An inter-island comparison of Darwin’s finches reveals the impact of habitat, host phylogeny, and island on the gut microbiome
Source: PLoS One. 2019 Dec 13;14(12):e0226432. doi: 10.1371/journal.pone.0226432 (PMC6910665; doi:10.1371/journal.pone.0226432)
Supplement: S4 Fig — Point color and point shape indicate host species and habitat, respectively. The four species that are present on both islands are plotted. A) Individual δ13C and δ15N values for each finch with gut microbiome samples. B) Mean δ13C and δ15N values for each species and habitat with standard deviation. Three points were the only sample from that habitat and species combination and therefore lack standard deviation error bars: the medium ground finch in the highlands and the small tree finch in the lowlands on Santa Cruz in addition to the cactus finch in the highlands on Floreana. (PDF) [file pone.0226432.s004.pdf]

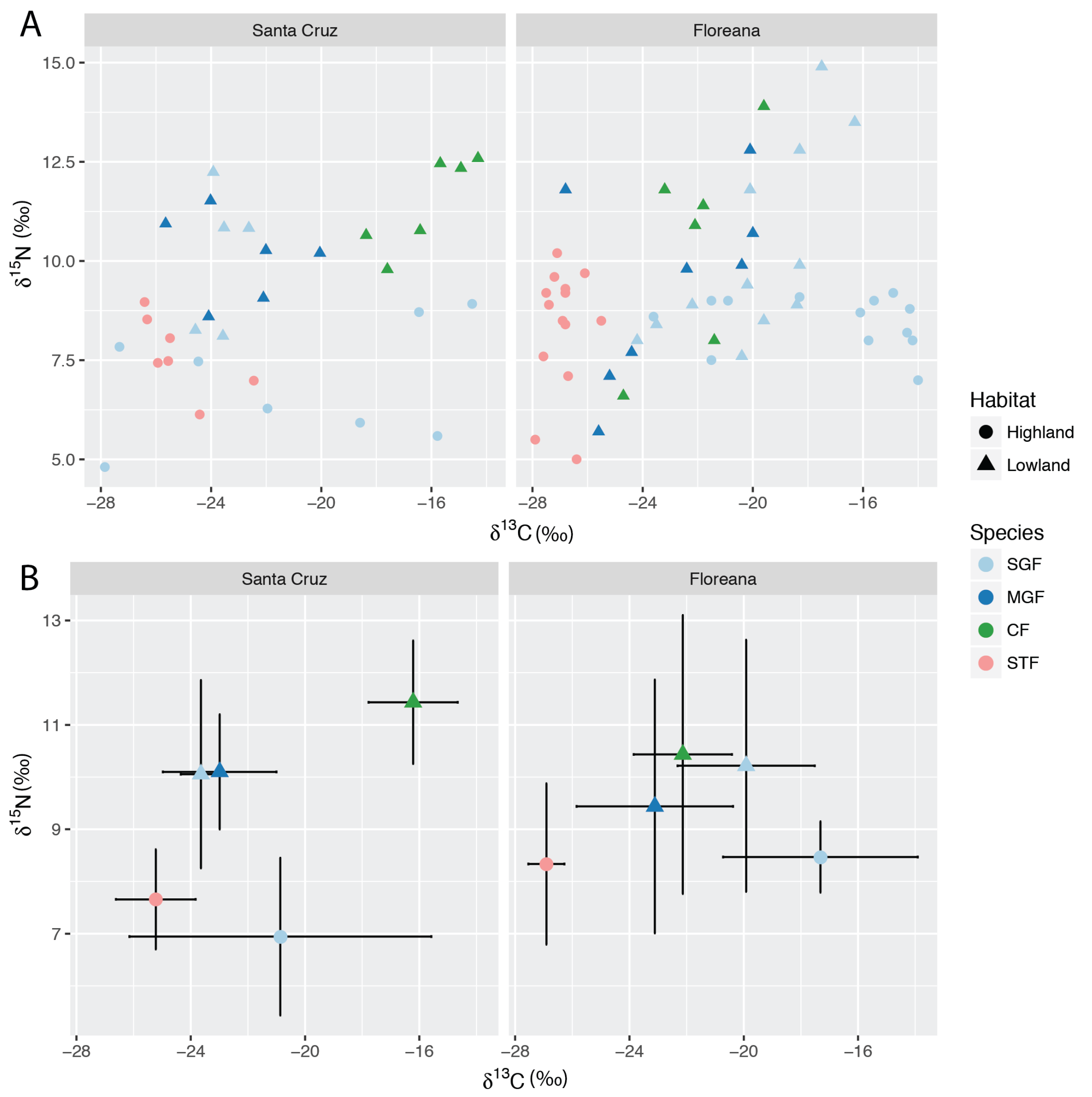

S4 Fig. δ^{13}C and δ^{15}N stable isotope measurements for Darwin's finch species across Santa Cruz and Floreana islands.

Point color and point shape indicate host species and habitat, respectively. The four species that are present on both islands are plotted. A) Individual δ^{13}C and δ^{15}N values for each finch with gut microbiome samples. B) Mean δ^{13}C and δ^{15}N values for each species and habitat with standard deviation. Three points were the only sample from that habitat and species combination and therefore lack standard deviation error bars: the medium ground finch in the highlands and the small tree finch in the lowlands on Santa Cruz in addition to the cactus finch in the highlands on Floreana.
