# Supplementary material for: An inter-island comparison of Darwin’s finches reveals the impact of habitat, host phylogeny, and island on the gut microbiome
Source: PLoS One. 2019 Dec 13;14(12):e0226432. doi: 10.1371/journal.pone.0226432 (PMC6910665; doi:10.1371/journal.pone.0226432)
Supplement: S5 Fig — Lines show pairwise Sorensen dissimilarities of Darwin’s finch gut microbiome samples determined by time slices every 10 Mya correlated to pairwise dietary distances calculated with first foraging observations (red), pairwise dietary distances using δ13C and δ15N stable isotope measurements (green), and pairwise host phylogenetic distances (blue). Correlation did not go above 0.15 for any of the three layers of metadata. (PDF) [file pone.0226432.s005.pdf]

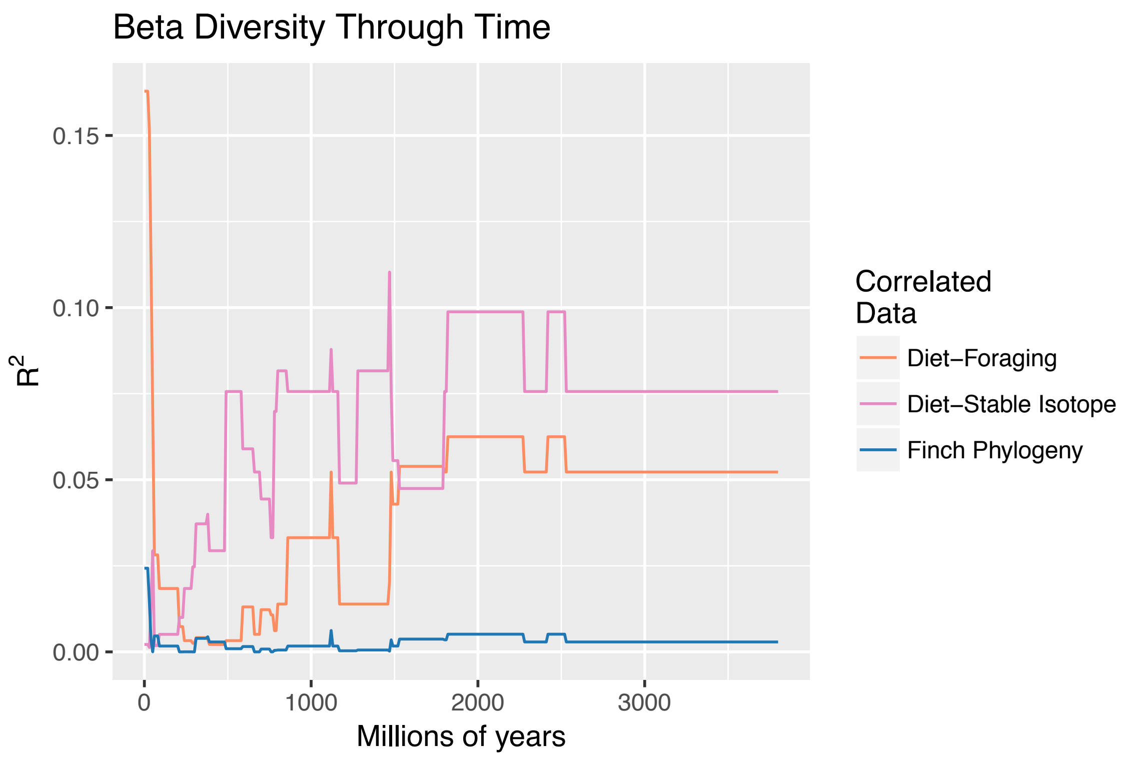

S5 Fig. Beta diversity through time applied on the gut microbiome data.

Lines show pairwise Sorensen dissimilarities of Darwin's finch gut microbiome samples determined by time slices every 10 Mya correlated to pairwise dietary distances calculated with first foraging observations (red), pairwise dietary distances using $\delta^{13}C$ and $\delta^{15}N$ stable isotope measurements (green), and pairwise host phylogenetic distances (blue). Correlation did not go above 0.15 for any of the three layers of metadata.
