# Supplementary material for: An inter-island comparison of Darwin’s finches reveals the impact of habitat, host phylogeny, and island on the gut microbiome
Source: PLoS One. 2019 Dec 13;14(12):e0226432. doi: 10.1371/journal.pone.0226432 (PMC6910665; doi:10.1371/journal.pone.0226432)
Supplement: S1 Table — (PDF) [file pone.0226432.s006.pdf]

S1 Table. Statistical tests on amplicon library size mean and distribution across categorical variables of interest

Variable	Kruskal-Wallis test for library size mean		Levene's test for library size distribution	
	X^2	p-value	F value	p-value
Species	2.79	0.73	0.40	0.85
Habitat	0.04	0.84	1.40	0.24
Sex	10.46	0.03	0.16	0.96
