# Supplementary material for: An inter-island comparison of Darwin’s finches reveals the impact of habitat, host phylogeny, and island on the gut microbiome
Source: PLoS One. 2019 Dec 13;14(12):e0226432. doi: 10.1371/journal.pone.0226432 (PMC6910665; doi:10.1371/journal.pone.0226432)
Supplement: S2 Table — (PDF) [file pone.0226432.s007.pdf]

S2 Table. Summary of samples used for inter-island comparison

Common Name	Abb.	Scientific Name	Floreana		Santa Cruz		Total
			H	L	H	L	
Small Ground finch	SGF	Geospiza fuliginosa	13	12	8	5	38
Medium Ground finch	MGF	Geospiza fortis		8		6	15
Cactus finch	CF	Geospiza scandens		6		6	12
Small Tree finch	STF	Camarhynchus parvulus	14		9		23
Total			27	26	17	17	87
