# Supplementary material for: An inter-island comparison of Darwin’s finches reveals the impact of habitat, host phylogeny, and island on the gut microbiome
Source: PLoS One. 2019 Dec 13;14(12):e0226432. doi: 10.1371/journal.pone.0226432 (PMC6910665; doi:10.1371/journal.pone.0226432)
Supplement: S3 Table — (PDF) [file pone.0226432.s008.pdf]

S3 Table. Relative abundance of bacterial phyla across all samples

Phylum	meanRA	sdRA	minRA	maxRA
Firmicutes	50.6%	34.7%	1.2%	99.5%
Actinobacteria	26.6%	26.4%	0.1%	96.0%
Proteobacteria	18.7%	18.1%	0.2%	77.1%
Unclassified	2.8%	9.2%	0.0%	55.3%
Chloroflexi	0.5%	0.6%	0.0%	2.8%
Acidobacteria	0.2%	0.5%	0.0%	2.8%
Planctomycetes	0.2%	0.2%	0.0%	1.2%
Chlamydiae	0.1%	1.1%	0.0%	9.5%
Cyanobacteria	0.1%	0.3%	0.0%	2.0%
Verrucomicrobia	0.0%	0.1%	0.0%	0.5%
Tenericutes	0.0%	0.1%	0.0%	0.8%
Deinococcus-Thermus	0.0%	0.0%	0.0%	0.3%
Bacteroidetes	0.0%	0.0%	0.0%	0.2%
