# Supplementary material for: An inter-island comparison of Darwin’s finches reveals the impact of habitat, host phylogeny, and island on the gut microbiome
Source: PLoS One. 2019 Dec 13;14(12):e0226432. doi: 10.1371/journal.pone.0226432 (PMC6910665; doi:10.1371/journal.pone.0226432)
Supplement: S4 Table — (PDF) [file pone.0226432.s009.pdf]

S4 Table. Relative abundance of bacterial phyla by Darwin finch species on Floreana Island

Phylum	SGF	MGF	CF	STF	HTF	MTF
Firmicutes	49.74%	50.28%	69.35%	42.10%	38.56%	88.86%
Actinobacteria	25.53%	36.01%	10.04%	30.94%	32.37%	7.52%
Proteobacteria	17.37%	11.94%	19.48%	24.96%	26.55%	2.90%
Unclassified	6.25%	0.40%	0.30%	1.07%	0.64%	0.60%
Chloroflexi	0.60%	0.55%	0.28%	0.35%	0.38%	0.02%
Acidobacteria	0.23%	0.37%	0.08%	0.30%	0.27%	0.02%
Planctomycetes	0.14%	0.21%	0.11%	0.18%	0.20%	0.03%
Chlamydiae	0.00%	0.00%	0.00%	0.00%	0.86%	0.00%
Cyanobacteria	0.04%	0.15%	0.29%	0.01%	0.04%	0.04%
Verrucomicrobia	0.02%	0.07%	0.05%	0.02%	0.03%	0.01%
Tenericutes	0.03%	0.00%	0.00%	0.00%	0.06%	0.00%
Deinococcus-Thermus	0.02%	0.01%	0.00%	0.05%	0.03%	0.00%
Bacteroidetes	0.03%	0.00%	0.00%	0.01%	0.03%	0.01%
Spirochaetes	0.00%	0.01%	0.00%	0.00%	0.00%	0.00%
