# Supplementary material for: An inter-island comparison of Darwin’s finches reveals the impact of habitat, host phylogeny, and island on the gut microbiome
Source: PLoS One. 2019 Dec 13;14(12):e0226432. doi: 10.1371/journal.pone.0226432 (PMC6910665; doi:10.1371/journal.pone.0226432)
Supplement: S5 Table — (PDF) [file pone.0226432.s010.pdf]

S5 Table. The relative abundance (%) of the most abundant bacterial genera across all Darwin finch gut microbiome samples from Floreana.

Phylum	Genus	meanRA	sdRA	minRA	maxRA
Firmicutes	Lactobacillus	43.55	36.51	0.05	99.41
Proteobacteria	Acinetobacter	5.39	12.82	0.00	54.67
Actinobacteria	Kocuria	4.93	12.46	0.00	64.21
Proteobacteria	Methylobacterium	3.21	5.21	0.00	25.91
Firmicutes	Enterococcus	2.98	12.57	0.00	92.11
Actinobacteria	Cellulomonas	2.02	4.64	0.00	31.93
Actinobacteria	Rubrobacter	1.48	10.05	0.00	88.42
Actinobacteria	Curtobacterium	1.39	3.83	0.00	23.93
Actinobacteria	Nocardioides	1.27	1.82	0.00	10.01
Actinobacteria	Solirubrobacter	1.11	1.69	0.00	8.64
Actinobacteria	Actinomycetospora	1.10	1.84	0.00	7.77
Actinobacteria	Pseudonocardia	1.04	2.18	0.00	12.04
