# Supplementary material for: An inter-island comparison of Darwin’s finches reveals the impact of habitat, host phylogeny, and island on the gut microbiome
Source: PLoS One. 2019 Dec 13;14(12):e0226432. doi: 10.1371/journal.pone.0226432 (PMC6910665; doi:10.1371/journal.pone.0226432)
Supplement: S6 Table — (PDF) [file pone.0226432.s011.pdf]

S6 Table. Relative abundance (%) of the most abundant bacterial genus in each species of Darwin's finches on Floreana

Genus	Species	meanRA	sdRA	minRA	maxRA
Lactobacillus	SGF	43.71	36.39	0.05	97.90
Lactobacillus	MGF	47.83	40.57	0.71	99.41
Lactobacillus	CF	48.15	35.37	0.23	96.71
Lactobacillus	STF	35.68	36.79	0.10	93.12
Lactobacillus	HTF	29.56	31.32	0.05	87.17
Lactobacillus	MTF	88.54	5.33	82.18	95.23
