# Supplementary material for: An inter-island comparison of Darwin’s finches reveals the impact of habitat, host phylogeny, and island on the gut microbiome
Source: PLoS One. 2019 Dec 13;14(12):e0226432. doi: 10.1371/journal.pone.0226432 (PMC6910665; doi:10.1371/journal.pone.0226432)
Supplement: S7 Table — (PDF) [file pone.0226432.s012.pdf]

S7 Table. Relative abundance (%) of the second most abundant bacterial genus in each species of Darwin's finches on Floreana

Genus	Species	meanRA	sdRA	minRA	maxRA
Acinetobacter	SGF	4.9	11.0	0.0	37.8
Kocuria	MGF	9.5	19.0	0.0	64.2
Enterococcus	CF	13.2	34.8	0.0	92.1
Kocuria	STF	11.9	20.5	0.0	62.3
Helicobacter	HTF	6.7	22.3	0.0	74.1
Kocuria	MTF	3.6	3.9	0.0	8.2
