# Supplementary material for: An inter-island comparison of Darwin’s finches reveals the impact of habitat, host phylogeny, and island on the gut microbiome
Source: PLoS One. 2019 Dec 13;14(12):e0226432. doi: 10.1371/journal.pone.0226432 (PMC6910665; doi:10.1371/journal.pone.0226432)
Supplement: S8 Table — (PDF) [file pone.0226432.s013.pdf]

S8 Table. Relative abundance of bacterial taxa in small ground finch samples from highland and lowland habitats on Floreana

Phylum	Highland	Lowland
Firmicutes	69.5%	39.9%
Proteobacteria	15.0%	18.2%
Actinobacteria	13.8%	38.9%
Unclassified	1.0%	1.3%
Chloroflexi	0.2%	1.0%
**Order**	**Highland**	**Lowland**
Lactobacillales	67.8%	38.6%
Actinomycetales	12.1%	28.6%
Pseudomonadales	8.8%	3.4%
Rhizobiales	4.3%	9.8%
Unclassified	1.3%	2.2%
Bacillales	1.3%	1.1%
Solirubrobacterales	1.2%	2.1%
**Genus**	**Highland**	**Lowland**
Lactobacillus	64.7%	38.4%
Acinetobacter	8.3%	3.2%
Unclassified	4.1%	7.7%
Curtobacterium	2.8%	1.0%
Methylobacterium	2.6%	2.0%
Weissella	1.7%	0.0%
Enterococcus	1.1%	0.1%
Staphylococcus	1.0%	0.1%
