# Supplementary material for: An inter-island comparison of Darwin’s finches reveals the impact of habitat, host phylogeny, and island on the gut microbiome
Source: PLoS One. 2019 Dec 13;14(12):e0226432. doi: 10.1371/journal.pone.0226432 (PMC6910665; doi:10.1371/journal.pone.0226432)
Supplement: S9 Table — (PDF) [file pone.0226432.s014.pdf]

S9 Table. Relative abundance of bacterial taxa in the small ground finch adults and nestlings from Floreana Island.

Phylum	Adult	Nestling
Firmicutes	39.9%	15.7%
Actinobacteria	38.9%	19.0%
Proteobacteria	18.2%	22.9%
Unclassified	1.3%	41.5%
Chloroflexi	1.0%	0.7%
Acidobacteria	0.3%	0.1%
Planctomycetes	0.2%	0.1%
**Class**	**Adult**	**Nestling**
Bacilli	39.7%	9.6%
Actinobacteria	38.9%	18.9%
Alphaproteobacteria	13.7%	6.3%
Gammaproteobacteria	4.1%	16.2%
Unclassified	1.9%	41.7%
**Genus**	**Adult**	**Nestling**
Lactobacillus	38.4%	0.6%
Rubrobacter	7.8%	0.5%
Unclassified	7.7%	59.2%
Cellulomonas	5.8%	2.6%
Acinetobacter	3.2%	0.1%
Paracoccus	2.3%	1.4%
Aurantimonas	2.0%	0.5%
Methylobacterium	2.0%	0.5%
