# Supplementary material for: An inter-island comparison of Darwin’s finches reveals the impact of habitat, host phylogeny, and island on the gut microbiome
Source: PLoS One. 2019 Dec 13;14(12):e0226432. doi: 10.1371/journal.pone.0226432 (PMC6910665; doi:10.1371/journal.pone.0226432)
Supplement: S10 Table — (PDF) [file pone.0226432.s015.pdf]

S10 Table. Alpha diversity estimates by Darwin's finch species on Floreana

Species	Observed ASVs mean	Observed ASVs SE	Chao1 mean	Chao1 SE
SGF	599.6	51.0	832.4	61.3
MGF	649.7	131.1	871.0	151.4
CF	536.7	139.7	785.8	171.0
STF	585.3	86.3	770.2	101.0
HTF	692.2	91.7	896.9	101.7
MTF	324.0	75.7	506.2	109.6
