# Supplementary material for: An inter-island comparison of Darwin’s finches reveals the impact of habitat, host phylogeny, and island on the gut microbiome
Source: PLoS One. 2019 Dec 13;14(12):e0226432. doi: 10.1371/journal.pone.0226432 (PMC6910665; doi:10.1371/journal.pone.0226432)
Supplement: S11 Table — (PDF) [file pone.0226432.s016.pdf]

S11 Table. Anova on alpha diversity metrics across species (N = 6, sample size ranging from 4 to 28)

Diversity metric	F value	p value
Observed ASVs	0.88	0.50
Chao 1	0.75	0.59
