# Supplementary material for: An inter-island comparison of Darwin’s finches reveals the impact of habitat, host phylogeny, and island on the gut microbiome
Source: PLoS One. 2019 Dec 13;14(12):e0226432. doi: 10.1371/journal.pone.0226432 (PMC6910665; doi:10.1371/journal.pone.0226432)
Supplement: S12 Table — +Age was tested using only small ground finch (G. fuliginosa) samples collected in the lowland (Adults = 12, Nestling = 3). (PDF) [file pone.0226432.s017.pdf]

S12 Table. Permanova tests of weighted UniFrac distances with categorical variables of interest

Variable	F	R^2	p-value
Habitat	8.24	0.10	0.001
Species	1.20	0.08	0.21
Habitat:Species	1.33	0.03	0.20
Sex	0.97	0.51	0.42
Age[+]	1.45	0.10	0.17

[+] Age was tested using only small ground finch (*G. fuliginosa*) samples collected in the lowland (Adults=12, Nestling=3)
