# Supplementary material for: An inter-island comparison of Darwin’s finches reveals the impact of habitat, host phylogeny, and island on the gut microbiome
Source: PLoS One. 2019 Dec 13;14(12):e0226432. doi: 10.1371/journal.pone.0226432 (PMC6910665; doi:10.1371/journal.pone.0226432)
Supplement: S13 Table — * Single sample for this species/habitat so standard deviation was not calculated. (PDF) [file pone.0226432.s018.pdf]

S13 Table. Stable isotope (δ ^{13}C and δ ^{15}N) ratios by Darwin's finch species and habitat on Floreana

Species	Habitat	δ ^{13}C mean (‰)	δ ^{13}C SD (‰)	δ ^{15}N mean (‰)	δ ^{15}N SD (‰)
SGF	H	-17.3	3.4	8.5	0.7
	L	-20.2	2.3	10.2	2.1
MGF	H	-27.4	1.0	8.7	2.2
	L	-23.1	2.7	9.4	2.4
CF	H	-23.0	*	8.5	*
	L	-22.1	1.7	10.4	2.7
STF	H	-26.9	0.6	8.3	1.5
HTF	H	-26.3	1.5	8.0	1.1
MTF	H	-26.5	1.2	8.6	0.5

* Single sample for this species/habitat so standard deviation was not calculated
