# Supplementary material for: An inter-island comparison of Darwin’s finches reveals the impact of habitat, host phylogeny, and island on the gut microbiome
Source: PLoS One. 2019 Dec 13;14(12):e0226432. doi: 10.1371/journal.pone.0226432 (PMC6910665; doi:10.1371/journal.pone.0226432)
Supplement: S14 Table — (PDF) [file pone.0226432.s019.pdf]

S14 Table. PERMANOVA results for combined dataset across Santa Cruz and Floreana Island with weighted UniFrac distances

Variable	Df	SumsOfSqs	MeanSqs	F.Model	R2	Pr(>F)
Island	1	0.03	0.03	1.9993	0.02	0.064
Habitat	1	0.21	0.21	12.4452	0.12	0.001
Island:Habitat	1	0.04	0.04	2.0768	0.02	0.048
Island:Habitat:Species	6	0.12	0.02	1.1826	0.07	0.202
Residuals	77	1.33	0.02		0.77	
Total	86	1.74				
