# Supplementary material for: An inter-island comparison of Darwin’s finches reveals the impact of habitat, host phylogeny, and island on the gut microbiome
Source: PLoS One. 2019 Dec 13;14(12):e0226432. doi: 10.1371/journal.pone.0226432 (PMC6910665; doi:10.1371/journal.pone.0226432)
Supplement: S15 Table — (PDF) [file pone.0226432.s020.pdf]

S15 Table. Post hoc pairwise comparisons of island and habitat combinations with weighted UniFrac distances

Pairwise comparison	Df	SumsOfSqs	MeanSqs	F.Model	R2	Pr(>F)
Highland Islands	1	0.11	0.11	3.18	0.07	0.008
Lowland Islands	1	0.04	0.04	0.90	0.02	0.431
Santa Cruz Habitats	1	0.19	0.19	7.31	0.19	0.001
Floreana Habitats	1	0.26	0.26	7.30	0.13	0.001
