# Supplementary material for: An inter-island comparison of Darwin’s finches reveals the impact of habitat, host phylogeny, and island on the gut microbiome
Source: PLoS One. 2019 Dec 13;14(12):e0226432. doi: 10.1371/journal.pone.0226432 (PMC6910665; doi:10.1371/journal.pone.0226432)
Supplement: S16 Table — (PDF) [file pone.0226432.s021.pdf]

S16 Table. Pairwise Euclidean distances between weighted average foraging patterns in each island/habitat combination for all five food categories (lower triangle) or broad plant v insect food categories (upper triangle).

	FL_High	FL_Low	SC_High	SC_Low
**FL_High**	-	0.84	0.18	0.88
**FL_Low**	0.71	-	0.67	0.03
**SC_High**	0.16	0.61	-	0.70
**SC_Low**	0.73	0.05	0.63	-
